# Mass spectrometry profiling of oxylipins, endocannabinoids, and *N*-acylethanolamines in human lung lavage fluids reveals responsiveness of prostaglandin E2 and associated lipid metabolites to biodiesel exhaust exposure

**DOI:** 10.1007/s00216-017-0243-8

**Published:** 2017-02-24

**Authors:** Sandra Gouveia-Figueira, Masoumeh Karimpour, Jenny A. Bosson, Anders Blomberg, Jon Unosson, Jamshid Pourazar, Thomas Sandström, Annelie F. Behndig, Malin L. Nording

**Affiliations:** 1 Department of Chemistry, Umeå University, 90187 Umeå, Sweden; 2 Department of Public Health and Clinical Medicine, Division of Medicine/Respiratory Medicine, Umeå University, 90187 Umeå, Sweden

**Keywords:** BAL, BW, Lipidome, Air pollution, Bronchoscopy, Eicosanoid

## Abstract

The adverse effects of petrodiesel exhaust exposure on the cardiovascular and respiratory systems are well recognized. While biofuels such as rapeseed methyl ester (RME) biodiesel may have ecological advantages, the exhaust generated may cause adverse health effects. In the current study, we investigated the responses of bioactive lipid mediators in human airways after biodiesel exhaust exposure using lipidomic profiling methods. Lipid mediator levels in lung lavage were assessed following 1-h biodiesel exhaust (average particulate matter concentration, 159 μg/m^3^) or filtered air exposure in 15 healthy individuals in a double-blinded, randomized, controlled, crossover study design. Bronchoscopy was performed 6 h post exposure and lung lavage fluids, i.e., bronchial wash (BW) and bronchoalveolar lavage (BAL), were sequentially collected. Mass spectrometry methods were used to detect a wide array of oxylipins (including eicosanoids), endocannabinoids, *N*-acylethanolamines, and related lipid metabolites in the collected BW and BAL samples. Six lipids in the human lung lavage samples were altered following biodiesel exhaust exposure, three from BAL samples and three from BW samples. Of these, elevated levels of PGE_2_, 12,13-DiHOME, and 13-HODE, all of which were found in BAL samples, reached Bonferroni-corrected significance. This is the first study in humans reporting responses of bioactive lipids following biodiesel exhaust exposure and the most pronounced responses were seen in the more peripheral and alveolar lung compartments, reflected by BAL collection. Since the responsiveness and diagnostic value of a subset of the studied lipid metabolites were established in lavage fluids, we conclude that our mass spectrometry profiling method is useful to assess effects of human exposure to vehicle exhaust.

**Electronic supplementary material:**

The online version of this article (doi:10.1007/s00216-017-0243-8) contains supplementary material, which is available to authorized users.

## Introduction

Air pollution contributes substantially to the global burden of respiratory and cardiovascular disease [1–4]. Numerous studies have shown a consistent association between particulate matter (PM) air pollution and respiratory morbidity and mortality [1, 5, 6]. Furthermore, controlled chamber exposure studies exploring cardiovascular and airway inflammatory responses to petrodiesel exhaust have shown induction of a neutrophil-dependent inflammation in the respiratory tract of healthy human subjects, as well as adverse effects on the cardiovascular system [7–21]. The underlying mechanisms of the induced airway inflammation have been described involving production of pro- and anti-inflammatory cytokines and chemokines, resulting in an influx of inflammatory cells to the lung. However, information is lacking on key molecules in this process: bioactive lipid mediators [22] with relevant roles in initiation, propagation, and resolution of inflammation [23, 24].

**Fig. 1:**
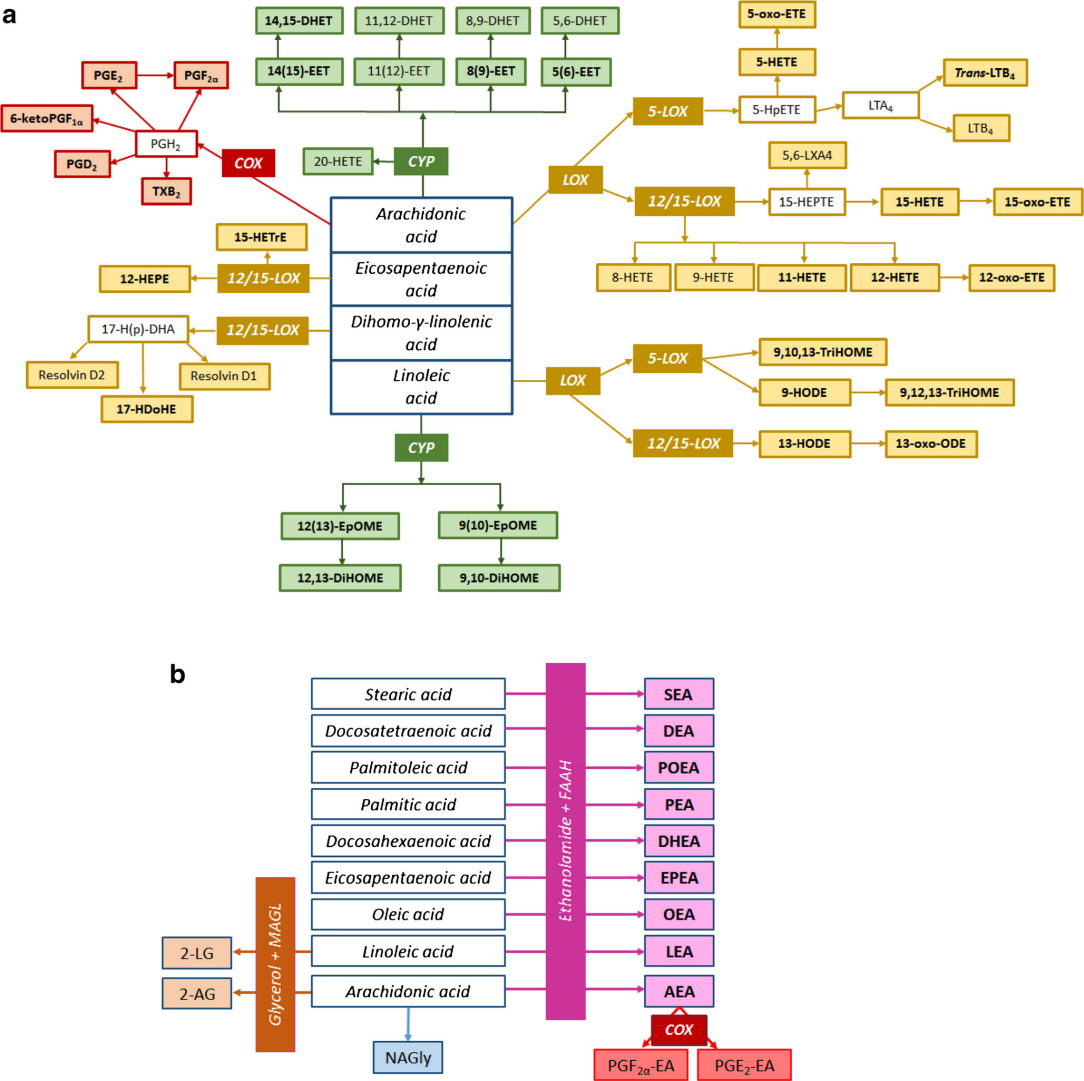
Scheme representing the oxylipins (**a**), and the endocannabinoids, *N*-acylethanolamines, and fatty acid glycerol esters (**b**) that were analyzed (in filled squares), with their enzymatic pathways. Detected metabolites in lung lavage fluids are highlighted in bold. Abbreviations: *COX*—cyclooxygenase, *LOX*—lipoxygenase, *CYP*—cytochrome P450, *FAAH*—fatty acid amide hydrolase, *MAGL*—monoacylglycerol lipase. Compounds from the COX pathway included prostaglandins (PGD_2_, PGE_2_, PGF_2α_, and 6-ketoPGF_1α_) and thromboxane (TXB_2_). The LOX pathway metabolites included hydroxyeicosatetraenoic acids (HETEs), hydroxyeicosapentaenoic acids (HEPEs), leukotrienes (LTB_4_), oxoeicosatetraenoic acids (oxo-ETEs), hydroxyeicosatrienoic acids (HETrEs), oxooctadecadienoic acid (oxo-ODEs), hydroxyoctadecadienoic acids (HODEs), and trihydroxyoctadecenoic acids (TriHOMEs). The CYP pathway included epoxyeicosatrienoic acids (EETs), epoxyoctadecenoic acids (EpOMEs), dihydroxyoctadecenoic acids (DiHOMEs), and dihydroxyeicosatrienoic acids (DHETs). Endocannabinoids, *N*-acylethanolamines, and fatty acid glycerol esters included the following: 2-linoleoylglycerol (*2-LG*), 2-arachidonoylglycerol (*2-AG*), *N*-arachidonylglycine (*NAGly*), stearoyl ethanolamide (*SEA*), docosatetraenyl ethanolamide (*DEA*), palmitoleoyl ethanolamide (*POEA*), palmitoyl ethanolamide (*PEA*), docosahexaenoyl ethanolamide (*DHEA*), eicosapentaenoyl ethanolamide (*EPEA*), oleoyl ethanolamide (*OEA*), linoleoyl ethanolamide (*LEA*), arachidonoyl ethanolamide (*AEA*), prostaglandin F_2α_ ethanolamide (*PGF*_*2α*_ *-EA*), and prostaglandin E_2_ ethanolamide (*PGE*_*2*_ *-EA*)

Oxylipins comprise a group of bioactive lipids mediating inflammatory events and they are biosynthesized on-demand through oxidation of polyunsaturated fatty acids (PUFA), for instance ω6 arachidonic acid (20:4n6), to produce eicosanoids such as prostaglandin E_2_ (PGE_2_), PGD_2_, PGF_2α_, and 15-hydroxyeicosatetraenoic acid (15-HETE). Oxylipins can also be produced from other PUFAs, a common one being linoleic acid (LA), resulting in compounds like 9-hydroxyoctadecadienoic acid (9-HODE), 13-HODE, 9,10-dihydroxyoctadecenoic acid (9,10-DiHOME), and 12,13-DiHOME. These oxylipins are formed via cyclooxygenase (COX), lipoxygenase (LOX), and cytochrome P450 (CYP) pathways (Fig. 1), as well as through non-enzymatic reactions, and target a wide variety of receptors [22, 24]. Another group of bioactive lipid mediators, the endocannabinoids, consists of ligands to the cannabinoid (CB) receptors, such as anandamide (AEA) and 2-arachidonoyl glycerol (2-AG) [25, 26]. In addition, there is an array of endocannabinoid-related lipids (*N*-acylethanolamines and glycerol fatty acid derivatives) with the ability to influence the activity of the CB_1_ and CB_2_ receptors [27], such as stearoyl ethanolamide (SEA) and palmitoyl ethanolamide (PEA).

Mapping this multifaceted network of interrelated fatty acid metabolic pathways by quantitative mass spectrometry (MS) coupled to liquid chromatography (LC) has proven useful, in various physiological contexts, for resolving the roles of participating bioactive lipid mediators [28, 29]. LC-MS/MS currently provides the most sensitive and specific analytical protocols for analyzing eicosanoids and other oxylipins as well as endocannabinoids and endocannabinoid-related compounds [30, 31]. In the present study, this technology was used to map bioactive lipid mediators in human lung lavage fluids from different regions of the lung (proximal and distal) which have not been fully explored before with regard to environmental exposure.

Due to diminishing oil reserves and environmental concern, considerable efforts have been made to find alternatives to petrodiesel, such as rapeseed methyl ester (RME) biodiesel, now among the most common biodiesel fuels in northern Europe. The use and combustion of biodiesel fuels are considered more ecologically beneficial than petrodiesel, as it is renewable, sustainable and has lower greenhouse gas emissions [32–35]. However, the effect of biodiesel exhaust exposure on human health has long been overlooked in spite of calls for such research, given the different emission profile compared to conventional fuels [36, 37].

In an exploratory study using a controlled experimental set-up, we have therefore previously investigated shifts in the metabolite profiles of the lungs following exposure to biodiesel exhaust compared to filtered air [38]. However, it is not known if the observed shifts also apply to specific constituents of the fatty acid metabolism, including inflammatory signaling molecules, i.e., oxylipins, endocannabinoids, and related lipid metabolites. In the current study, we therefore tested the hypothesis that oxylipin and related lipid metabolite profiles in the human lung shift in response to exposure to exhaust generated from RME biodiesel fuel in its pure form (B100). As a secondary aim, different lipid metabolite profiles in distinct regions of the lung were investigated.

## Materials and methods

### Chemicals and solvents

Most native and deuterated standards for analysis of oxylipins, endocannabinoids, and endocannabinoid-related lipids, including 12-[[(cyclohexylamino)carbonyl]amino]-dodecanoic acid (CUDA) used for recovery calculation purposes, were obtained from Cayman Chemical (Ann Arbor, MI, USA). Only 9,10,13-trihydroxyoctadecenoic acid (TriHOME) and 9,12,13-TriHOME were obtained from Larodan (Malmö, Sweden). Acetonitrile (ACN) and methanol (MeOH) were from Merck (Darmstadt, Germany). Isopropanol was from VWR PROLABO (Fontenay-sous-Bois, France). Acetic acid was purchased from Aldrich Chemical Company, Inc. (Milwaukee, WI, USA). Butylhydroxytoluene (BHT) was from Cayman Chemical (Ann Arbor, MI, USA) and ethylenediaminetetraacetic acid (EDTA) from Fluka Analytical, Sigma-Aldrich (Buchs, Switzerland). Glycerol was from Fischer Scientific (Loughborough, UK). All solvents and chemicals were of HPLC grade or higher and ultrapure water was used (Milli-Q Gradient system, Millipore, Milford, MA, USA).

### Study design

The study design was based on a series of preceding studies, as previously outlined [18]. In short, the subjects were healthy never smokers and underwent routine health examination including respiratory function. Fifteen individuals with the following characteristics were included in the crossover study design: 8 males, 7 females (mean age 26 years, range 19–34 years); mean body mass index 22 kg/m^2^ (range 20–26 kg/m^2^). Each subject was instructed to have a light, ordinary breakfast, to avoid ham, and to eat as similarly as possible before the two exposures. Subjects were also instructed to refrain from alcohol and caffeine for 24 h pre-exposure, and to abstain from use of anti-inflammatory drugs or dietary supplements during the week preceding the exposure and bronchoscopy.

**Fig. 2:**
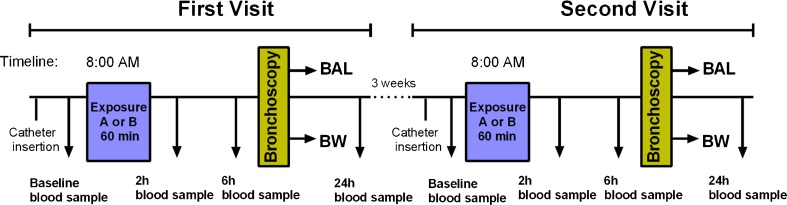
The study design, including sampling time points. At the first visit, the subjects were randomized to either exposure *A* (biodiesel exhaust) or *B* (sham exposure using filtered air). Bronchial wash (*BW*) and bronchoalveolar lavage (*BAL*) were collected by bronchoscopy around 6 h after completion of the 1-h exposure session. At the second visit (at least 3 weeks later), the subjects were crossed over to the other type of exposure and underwent the same sampling protocol

Each subject underwent two exposures, with a minimum 3-week interval (Fig. 2). Each 1-h exposure session started around 8 a.m., during which the subjects were exposed to either filtered air or biodiesel exhaust with an average PM concentration of 159 μg/m^3^ in a human exposure chamber in a controlled randomized, double-blinded, crossover fashion. Biodiesel exhaust emissions were generated from RME in its pure form (B100) by a Volvo engine (Volvo TD40 GJE, 4.0 L, four cylinders) using the urban part of the European Transient Cycle, in order to mimic urban driving conditions [14]. The controlled environment in the chamber was continuously monitored for particulate number and concentration as well as various pollutant gases. During the exposure, subjects alternated 15-min intervals of exercise, at an average minute ventilation of 20 L/m^2^ body surface area, with rest. The bronchoscopy procedure was performed around 6 h after completion of the exposure session, according to a well-established, previously described method [18]. The time point for the bronchoscopy procedure was chosen based on previous findings on the elevated inflammatory response in the airways at 6 h after exposure [18]. A detailed description of the method is provided in the Electronic supplementary material (ESM). Briefly, two 20-mL volumes followed by three 60-mL volumes of sterile saline solution were infused into either the right middle or left lingual lobe of the lungs, and gently suctioned back to sequentially collect bronchial wash (BW) and bronchoalveolar lavage (BAL), respectively. All samples were stored at −80 °C until analysis. The subjects were allowed to have a light breakfast in the morning, prior to the exposure, but were fasting thereafter until 2 h after the bronchoscopy procedure.

### Combined sample extraction for lipid metabolites

A previously reported solid phase extraction (SPE) protocol [31] was modified to isolate oxylipins, endocannabinoids, *N-*acylethanolamines, and other related lipids from BW and BAL samples. In summary, extraction was performed on Waters Oasis HLB cartridges (60 mg of sorbent, 30 μm particle size). These were first washed with ethyl acetate (1 mL) and MeOH (2 × 2 mL), then conditioned with 5% MeOH in water (containing 0.1% acetic acid) before loading the sample. Quantitative volumes from each sample (resulting in ∼6 mL BAL and 1–3 mL BW) were spiked with 10 μL internal standard (IS) mixture in methanol containing 50 ng/mL 12,13-DiHOME-d_4_ and 12(13)-EpOME-d_4_, 25 ng/mL 9(S)-HODE-d_4_, PGE_2_-d_4_ and TXB_2_-d_4_, 800 ng/mL 2-AG-d_8_, 40 ng/mL PGF_2α_-EA-d_4_ and PGE_2_-EA-d_4_, 20 ng/mL AEA-d_8_, OEA-d_4_, SEA-d_3_ and PEA-d_4_, as well as 10 μL antioxidant solution (0.2 mg/mL BHT/EDTA in methanol/water (1:1)). After applying the sample, IS, and antioxidant solution, the SPE cartridge was washed, dried under high vacuum and eluted with 3 mL acetonitrile, 2 mL methanol, and 1 mL ethyl acetate. All three fractions were pooled into polypropylene tubes containing 6 μL of a glycerol solution (30% in methanol) to trap the analytes. Eluates were evaporated using a MiniVac system (Farmingdale, NY, USA), reconstituted in 100 μL of MeOH, spiked with 10 μL recovery standard (50 ng/mL CUDA), transferred to vials, and analyzed by LC-MS/MS.

### LC-MS/MS equipment for analysis of lipid metabolites

The Agilent Ultra-Performance LC system (Infinity 1290) coupled to an Agilent 6490 Triple Quadrupole mass spectrometer with an electrospray ionization source (ESI) equipped with the iFunnel Technology (Agilent Technologies, Santa Clara, CA, USA) was used. Samples for ionization in positive (endocannabinoid-related lipids) and negative (oxylipins) mode were injected separately. Chromatographic separation was performed on a Waters BEH C_18_ column (2.1 mm × 150 mm, 130 Å, 1.7 μm particle size), with an injection volume of 10 μL for each run. The eluents in the mobile phase consisted of (A) deionized water (0.1% acetic acid) and (B) acetonitrile/isopropanol (90:10). For oxylipin separation, the following gradient was employed: 0.0–3.5 min 10–35% B, 3.5–5.5 min 35–40% B, 5.5–7.0 min 40–42% B, 7.0–9.0 min 42–50% B, 9.0–15.0 min 50–65% B, 15.0–17.0 min 65–75% B, 17.0–18.5 min 75–85% B, 18.5–19.5 min 85–95% B, 19.5–21.0 min 95–10% B, 21.0–25.0 min 10% B [31]. The separation gradient for endocannabinoids and related lipids (including prostamides) was as follows: 0.0–2.0 min 30–45% B, 2.0–2.5 min 45–79% B, 2.5–11.5 min 79% B, 11.5–12.0 min 79–90% B, 12.0–14.0 min 90% B, 14.0–14.5 min 90–79% B, 14.5–15.5 min 79% B, 15.6–19.0 min 30% B [31]. The last 3 min of the gradient was directed to the waste to reduce MS contamination.

The ESI-MS/MS conditions were as follows: capillary and nozzle voltage at 4000 and 1500 V, drying gas temperature 230 °C with a gas flow of 15 L/min, sheath gas temperature 400 °C with a gas flow of 11 L/min; the nebulizer gas flow was 35 psi, and iFunnel high and low pressure RF were set at 90 and 60 V (negative mode) and 150 and 60 V (positive mode). Dynamic multiple reaction monitoring (MRM) mode was used with fixed time windows (retention time ±2 min) to profile two transitions per compound (one quantitative and one qualitative), see Tables S1 and S2 in the ESM. The dynamic MRM option was performed for all compounds with optimized transitions and collision energies. The MassHunter Workstation software was used for instrument control and for manual integration of all peaks.

### Standards and calibration curve preparation

The stable isotope dilution method was used to quantify the analytes. Two types of internal standard were used: (i) for quantification purposes, deuterated IS was added before extraction, and (ii) for monitoring the loss of IS, the recovery standard CUDA was added after extraction [39]. The addition of a known concentration of CUDA after sample preparation facilitated the assessment of IS recovery by providing a standard not affected by losses during extraction to normalize the IS peak area against. Five IS were used for quantification of endocannabinoids, *N*-acylethanolamines, and related lipids (AEA-d_8_, SEA-d_3_, PEA-d_4_, OEA-d_4_ and 2-AG-d_8_), and eight for oxylipin quantification (12,13-DiHOME-d_4_, 12(13)-EpOME-d_4_, 9-HODE-d_4_, PGE_2_-d_4_, PGD_2_-d_4_, 5-HETE-d_8_, 20-HETE-d_6_ and TXB_2_-d_4_). For each native compound, a suitable IS was selected based on structural similarities (Table S3, ESM). Standard solutions were prepared at 10 different levels (Tables S4 and S5, ESM) to determine calibration curves by the least-squares linear regression model with equal weighting factor, using the equation *y* = *mx* + *b*, where *y* corresponds to the response ratio (native standard peak area/internal standard peak area), *m* is the slope of the curve, *x* corresponds to the on-column concentration of the analyte, and *b* is the y-interception of the calibration curve.

### Statistical methods

Basal statistical measures (D’Agostino and Pearson omnibus normality tests and Wilcoxon matched-pairs signed rank test) and receiver-operating characteristic (ROC) curves were performed using the statistical package built into the Graphpad Prism software (version 6, GraphPad Software Inc., San Diego, CA, USA). For analytes below limit of detection (LOD), i.e., non-detects, half of the LOD value was used in the univariate statistical calculations [40]. Bonferroni correction for multiple testing was performed to avoid false positive results. Multivariate analysis using principal component analysis (PCA) and orthogonal projections to latent structures (OPLS) with discriminant analysis (DA) was done using the SIMCA software (Version 14, Umetrics, Umeå, Sweden) with raw data as input to investigate the relationship of lipid profiles with (i) exposure type (filtered air vs biodiesel exhaust) and (ii) lung lavage type (BW vs BAL). All data were mean-centered and scaled to unit variance before modeling.

ROC curves were generated to further investigate these findings by evaluating the oxylipins and *N*-acylethanolamines as diagnostic markers for exhaust exposure. ROC curves are non-parametric and consider true negatives (specificity) and true positives (sensitivity) identified at a given cutoff value of the feature under analysis, commonly used in metabolomics studies to avoid misinterpretation of the data [41]. The area under the curve (AUC) of a plot of the sensitivity vs 1 minus the specificity (1-specificity) indicates the diagnostic value of each feature analyzed. The maximum value for AUC is 1, and a value of 0.5 indicates no diagnostic value. The closer the AUC is to 1, the better the diagnostic usefulness of a specific feature. The optimal threshold value, called the Youden score, can be determined to assess the cutoff point of the diagnostic marker.

## Results

In this double-blinded, randomized, controlled, crossover study, lipid mediator levels in lung lavage were assessed in 15 healthy individuals, following a 1-h biodiesel exhaust (average PM 159 μg/m^3^) or filtered air (sham) exposure. Exposures were performed at two different occasions and in random order with subjects acting as their own controls. Six hours after the end of the exposure, bronchoscopy collection of BW and BAL was performed. Both the exposures and the bronchoscopies were well tolerated by all subjects. Our validated mass spectrometry methods [31] were used to detect a wide array of oxylipins, endocannabinoids, *N*-acylethanolamines, and fatty acid glycerol esters in the collected BW and BAL samples (raw data given in ESM, Table S6).

### Oxylipin levels

The oxylipin profiling method screened 41 compounds in the samples analyzed (Table S1, ESM). Of these, 21 were present at levels above the limit of quantification (LOQ) in at least 75% of either BW or BAL samples, and therefore included in univariate statistical analysis (Table 1).

**Table 1:** Oxylipin and *N*-acylethanolamine concentrations (pM) in bronchoalveolar lavage (BAL) and bronchial wash (BW) after filtered air or biodiesel exhaust (BioDE) exposure. Significant differences (Wilcoxon test with *α* = 0.05) are marked in bold and significant ones after Bonferroni correction (*P* value <0.002) are italicized. A comparison was made with literature data on lipid concentrations when present [42, 44]

	BAL							BW							BAL vs BW *P value*				
	Filtered air			BioDE			*P value*	Filtered air			BioDE			*P value*	Filtered air	BioDE	Ref [42]		Ref [44]
	Median	IQR	Range	Median	IQR	Range		Median	IQR	Range	Median	IQR	Range				BAL	BW	BAL
Oxylipins																			
Arachidonic acid (20:4n6) derivatives (*n* = 11)																			
TXB_2_	5.6	7.5	2.2–19	6.8	10	2.1–49	0.6	12	11	3.5–36	17	9.5	0–55	0.1	**0.04**	0.2			64
6-ketoPGF_1α_^a^	*–*	*–*	*–*	*–*	*–*	*–*	*n.a.*	4.1	4.4	0–15	3.8	1.2	1.8–14	0.2	*n.a.*	*n.a.*			
PGF_2α_	13	19	0–81	10	10	0–41	0.2	21	21	0–48	12	18	0–45	**0.04**	0.2	0.4			
PGE_2_^a^	1.6	1.4	0–3.7	3.5	3.9	0–6.7	***0.0006***	–	–	–	–	–	–	*n.a.*	*n.a.*	*n.a.*			10
PGD_2_^a^	5.6	6.2	1.5–11	3.7	4.5	0–21	0.6	–	–	–	–	–	–	*n.a.*	*n.a.*	*n.a.*			15
15-HETE	30	26	11–57	23	95	6.7–274	0.5	271	205	85–602	204	75	8.9–409	**0.03**	***<0.0001***	***<0.0001***	5.6	180	672
15-oxo-ETE	6.0	8.0	0–16	11	15	0–81	0.2	27	29	0–69	18	10	0–47	0.2	***0.002***	0.1			
12-HETE	14	8.6	0–22	14	39	0–165	0.2	55	107	21–248	51	58	18–123	0.3	***0.0005***	***0.001***	0	57	64
12-oxo-ETE^a^	*–*	–	–	–	–	–	*n.a.*	38	85	0–394	91	59	0–341	0.6	*n.a.*	*n.a.*			
5-HETE^a^	*–*	–	–	–	–	–	*n.a.*	9.0	13	0–19	3.4	15	0–31	>0.999	*n.a.*	*n.a.*			199
14(15)-EET^a^	*–*	–	–	–	–	–	*n.a.*	17	23	0–45	16	17	0–64	0.9	**0.02**	**0.008**			
Linoleic acid (18:2n6) derivatives (*n* = 8)																			
9,12,13-TriHOME	52	19	28-103	62	19	44–89	0.3	127	23	71–215	119	9.2	70–207	0.5	***<0.0001***	***<0.0001***	262	58.3	446
9,10,13-TriHOME	34	12	18-65	36	13	27–52	0.2	61	29	41–139	75	12	42–130	0.8	***0.0001***	***<0.0001***	388	58.3	262
12,13-DiHOME	43	44	19-99	66	40	42–127	***0.002***	38	24	19–89	35	5.4	21–81	0.9	0.1	***<0.0001***	67.7	137	476
9,10-DiHOME	41	35	17-91	55	36	27–103	0.2	27	12	12–63	26	1.7	17–35	0.4	**0.02**	***<0.0001***	63	44.7	408
13-HODE	164	78	103-358	310	138	164–1728	***0.002***	626	288	208–1085	552	163	361–848	0.2	***<0.0001***	0.06	178	343	1016
9-HODE	49	24	26-152	61	39	0–269	0.6	126	67	54–255	163	58	83–254	**0.05**	***0.002***	**0.007**	150	122	202
12(13)-EpOME	88	55	54-312	112	43	69–223	0.5	35.3	37	0–92	36	36	19–326	0.7	***0.0009***	**0.008**	657	340	1938
9(10)-EpOME	227	151	73-695	214	93	133–570	0.8	74	61	3.6–165	61	124	18–1196	0.3	***0.0002***	**0.02**	627	301	1877
Docosahexaenoic acid (22:6n3) derivative (*n* = 1)																			
17-HDoHE^a^	*–*	–	–	–	–	–	*n.a.*	95	83	0–172	97	82	0–228	0.7	*n.a.*	*n.a.*			278
Dihomo-γ-linolenic acid (20:3n6) derivative (*n* = 1)																			
15-HETrE	2.4	2.1	0–7.2	3.7	13	0–17	0.05	31	28	0–54	26	16	0–53	0.1	***0.0001***	***0.002***	0.8	16.9	63
*N*-acylethanolamines (*n* = 5)																			
OEA	43	22	23–87	49	15	33–81	0.2	94	32	59–265	86	9.5	51–157	0.4	***<0.0001***	***<0.0001***			
PEA	124	27	91–225	128	64	108–258	0.4	284	121	175–581	279	73	159–571	0.5	***<0.0001***	***<0.0001***			
POEA	34	15	25–52	37	6.3	29–50	0.7	79	37	47–341	82	36	52-235	0.5	***<0.0001***	***<0.0001***			
LEA^a^	–	–	–	–	–	–	–	11	22	0–159	19	17	6.4–154	0.6	***0.002***	***<0.0001***			
SEA	159	47	102-293	175	59	116–337	0.6	302	120	194–803	295	67	161–651	0.3	***<0.0001***	***0.002***			

^a^Detected in less than 75% of either BW or BAL samples (“–”)

*n.a.* not applicable

**Fig. 3:**
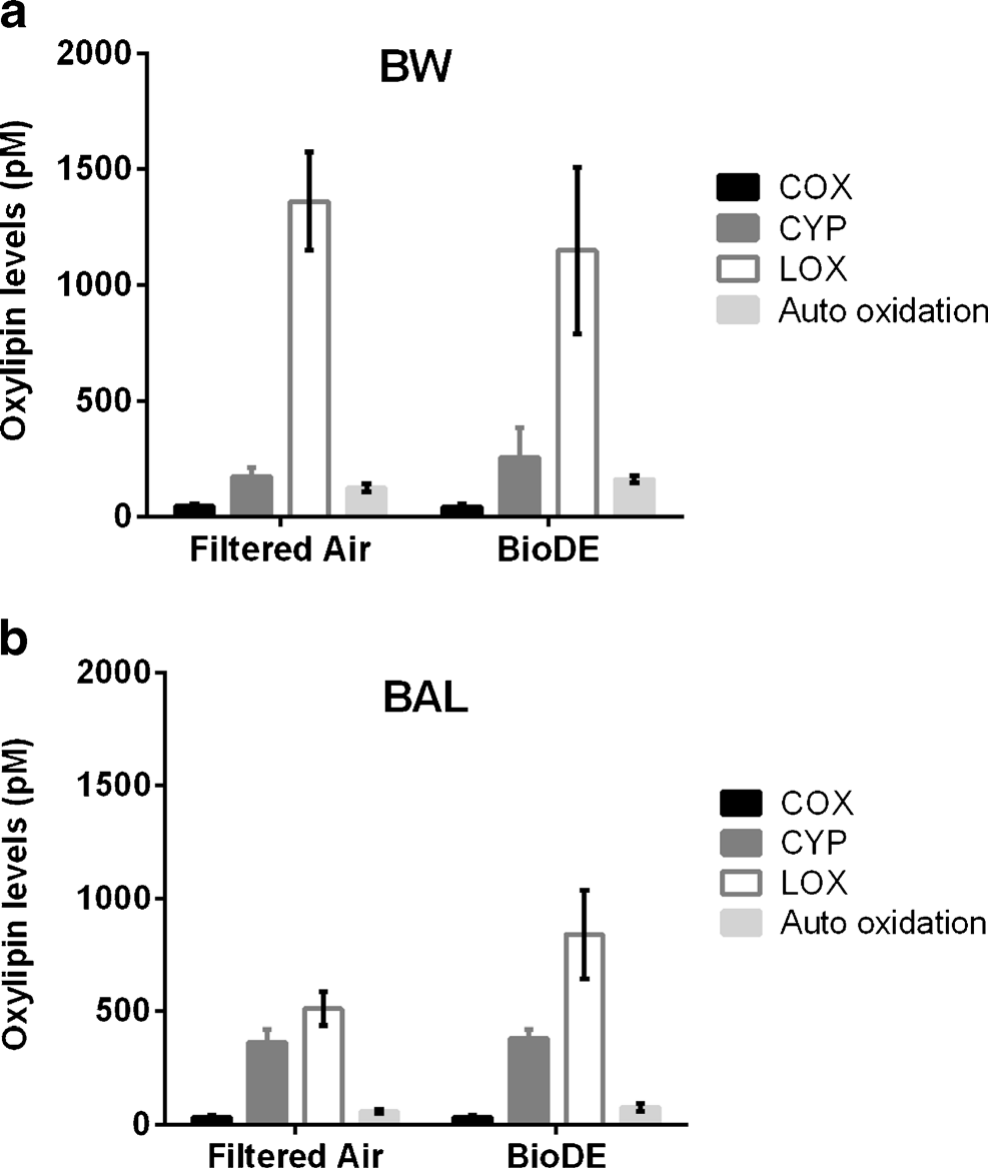
Total oxylipin concentrations (pM) in human **a** bronchial wash (*BW*) and **b** bronchoalveolar lavage (*BAL*) after exposure to filtered air and biodiesel exhaust (*BioDE*). Black bars represent cyclooxygenase (*COX*) derivatives, white bars represent lipoxygenase (*LOX*) pathway derivatives, dark gray bars represent cytochrome P450 (*CYP*) derivatives, and light gray bars represent auto oxidation products

Panels a and b of Fig. 3 present the total oxylipin concentrations (based on raw data) after each exposure in BW and BAL, respectively, according to their enzymatic pathway (Table S1, ESM). LOX products dominated the oxylipin profile, together with CYP metabolites (around 50% of the content for each pathway) in BAL, while LOX metabolites alone were present at the highest relative concentrations in BW (around 75%). Furthermore, BW contained higher levels of oxylipins than BAL, in agreement with Larsson et al. [42]. COX products constituted only 5 of 41 oxylipins in the analytical panel and thereby comprised the smallest fraction of the compounds detected.

### Levels of Endocannabinoids and *N*-acylethanolamines

Of the 14 compounds screened in the endocannabinoid metabolome (Table S2, ESM) using our validated LC-MS/MS protocol, four (OEA, SEA, PEA, and POEA) were present in all BW and BAL samples and LEA was detected in 90% of the BW samples, therefore these five *N*-acylethanolamines were included in univariate statistical analysis (Table 1). AEA and EPEA were present in only a few samples and were therefore not subjected to further statistical analysis. Hence, in total seven compounds were detected in BW and/or BAL, including AEA and six other *N*-acylethanolamines (no glycerol fatty acid derivatives were detected). Endocannabinoids and other related lipids (*N*-acylethanolamines and glycerol fatty acid derivatives) have not previously been reported in human BW or BAL, except in a study by Zoerner et al. [43], in which AEA was elevated in BAL from asthmatics following allergen challenge. We found concentrations in the range of 1.1–803 pM in the human lung lavage fluid, with PEA and SEA being the most abundant (Table 1 and ESM Table S6).

### Responsiveness of oxylipins and *N*-acylethanolamines from lung lavage fluids after biodiesel exhaust exposure

No compound passed the D’Agostino and Pearson omnibus normality test, even with square root transformation. Shifts in oxylipin and *N*-acylethanolamine levels due to biodiesel exhaust exposure were assessed using the Wilcoxon matched-pairs signed rank test and the results are shown in Table 1.

**Fig. 4:**
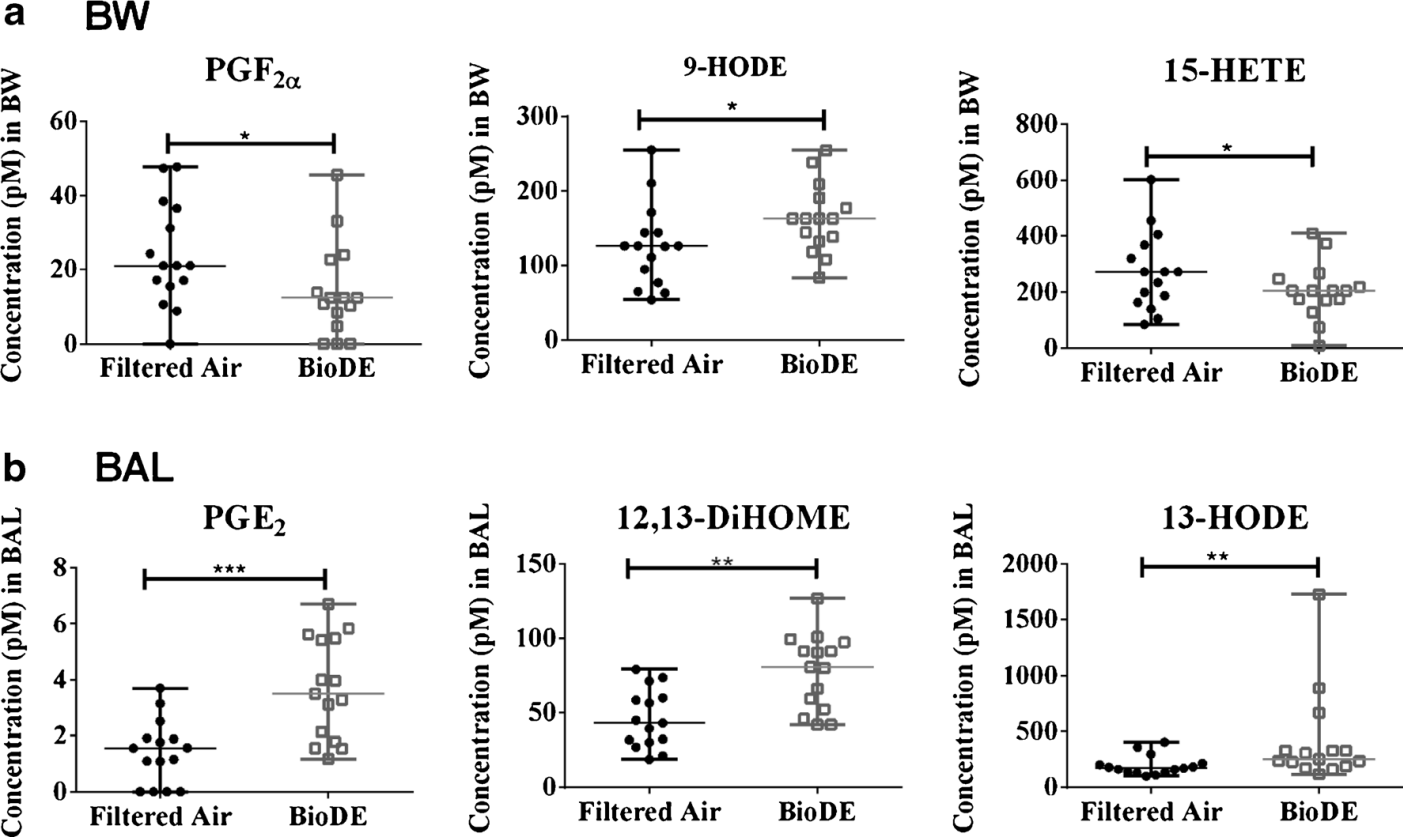
Concentrations of oxylipins (pM) in human **a** bronchial wash (*BW*) samples and **b** bronchoalveolar lavage (*BAL*) samples that were significantly altered due to biodiesel exhaust exposure (*BioDE*). Data are presented as median and interquartile range. Significance determined using the Wilcoxon test, and *P* values are summarized in Table 1 (*P < 0.05, **P < 0.01, ***P < 0.001)

In BW, decreased levels of PGF_2α_ (AA derivative, COX pathway) and 15-HETE (AA derivative, LOX pathway), as well as elevated levels of 9-HODE (LA derivative, LOX pathway) were associated with biodiesel exhaust exposure (Fig. 4a). In BAL, another three oxylipins displayed significant shifts following biodiesel exhaust exposure 12,13-DiHOME and 13-HODE (LA derivatives, CYP and LOX pathway, respectively) and PGE_2_ (AA derivative, COX pathway), see Fig. 4b. All of the analytes in Fig. 4a and Fig. 4b displayed a sufficient number of quantified values to support the validity of replacing non-detected values with LOD/2 in the statistical analysis [40]. All were present in 100% of BW or BAL samples, except PGF_2α_ (10% non-detected values) and PGE_2_ (13% non-detected values). Among the oxylipins and *N*-acylethanolamines, there were up to 26 possible comparisons, which corresponded to a threshold significance value of 0.002 (Bonferroni correction for multiple testing). All significant compounds in BAL (PGE_2_, 12,13-DiHOME and 13-HODE), but none in BW, reached this level (Table 1).

**Fig. 5:**
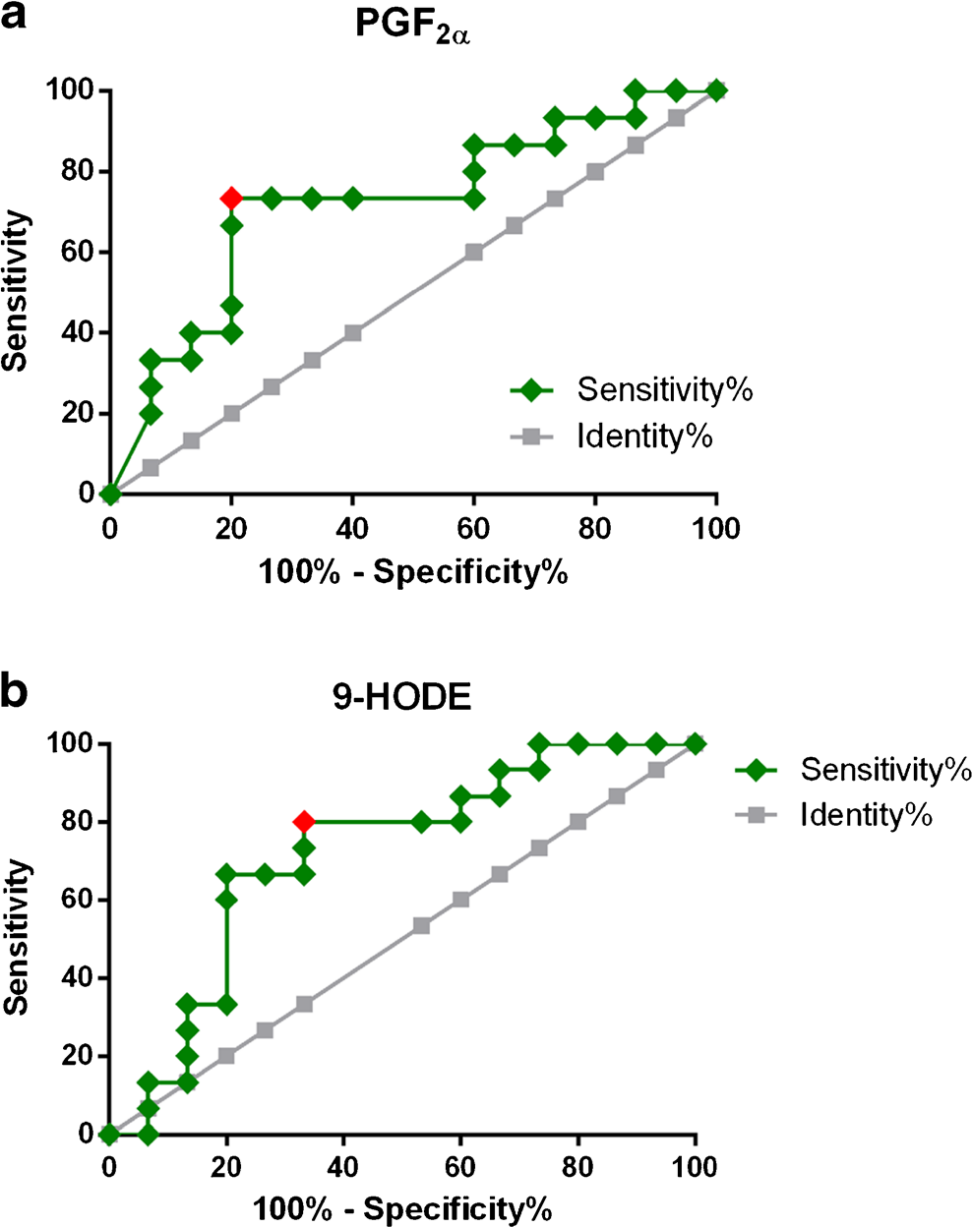
Receiver-operating characteristic (ROC) curves for oxylipins in bronchial wash (BW) samples. **a** PGF_2α_ (AUC = 0.72, 95% CI 0.53–0.91, *P* = 0.038; Youden score <16.2 pM); **b** 9-HODE (AUC = 0.72, 95% CI 0.53–0.91, *P* = 0.040; Youden score >129 pM)

**Fig. 6:**
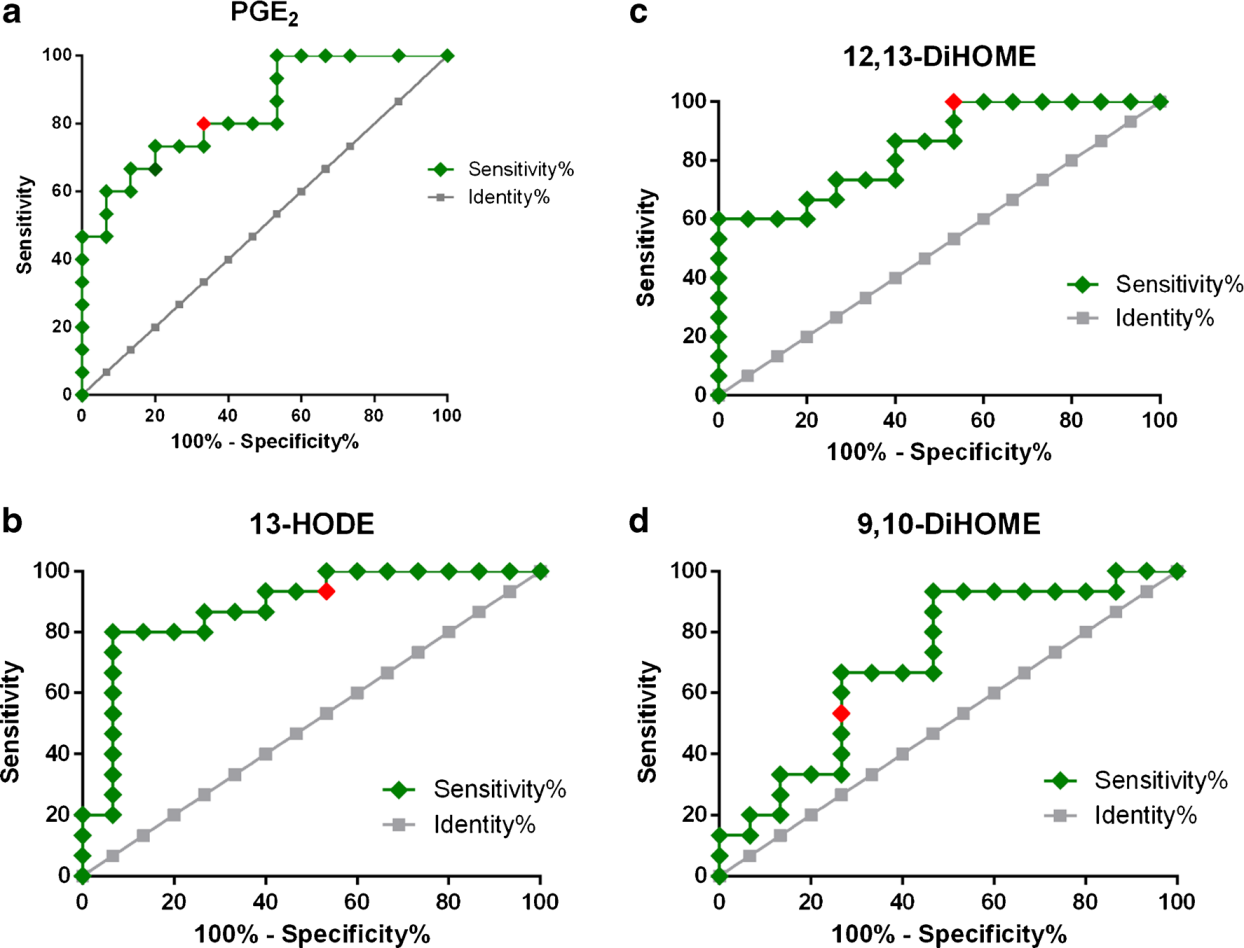
Receiver-operating characteristic (ROC) curves for oxylipins in bronchoalveolar lavage (BAL) samples that shifted significantly due to biodiesel exhaust exposure. **a** PGE_2_ (AUC = 0.75; 95% CI 0.57–0.93, *P* = 0.001; Youden score >3.22 pM); **b** 13-HODE (AUC = 0.76, 95% CI 0.58–0.94, *P* = 0.0015; Youden score >239 pM); **c** 12,13-DiHOME (AUC = 0.76, 95% CI 0.58–0.93, *P* = 0.010; Youden score >40.7 pM) and **d** 9,10-DiHOME (AUC = 0.71, 95% CI 0.52–0.89, *P* = 0.044; Youden score >42.3 pM)

We determined the AUC for ROC curves of all metabolites detected in BW and BAL and found that metabolites in BAL that had shifted significantly were far from reaching significance in BW and vice versa. The significant ROCs for metabolites in BW, PGF_2α_ (AUC = 0.72, 95% CI 0.53–0.91, *P* = 0.038) and 9-HODE (AUC = 0.72, 95% CI 0.53–0.91, *P* = 0.040), are shown in Fig. 5. The Wilcoxon test also determined that these compounds had shifted significantly (Fig. 4a). In BAL, four oxylipins displayed significant AUC values (Fig. 6), largely corroborating the outcome of the Wilcoxon test (Fig. 4b): PGE_2_ (AUC = 0.75, 95% CI 0.57–0.93, *P* = 0.001), 13-HODE (AUC = 0.76, 95% CI 0.58–0.94, *P* = 0.015), 12,13-DiHOME (AUC = 0.76, 95% CI 0.58–0.93, *P* = 0.010), and 9,10-DiHOME (AUC = 0.71, 95% CI 0.52–0.89, *P* = 0.044). However, 9,10-DiHOME displayed a *P* value close to the upper limit of significance (*α* = 0.05) and was not significant according to the Wilcoxon test. In Fig. 5 and Fig. 6, the Youden score for each compound is marked in red; for example, the COX metabolite PGE_2_ had a Youden score of >3.22 pM. When analyzing the concentrations of each sample, we found that BAL samples collected after biodiesel exhaust exposure produced PGE_2_ levels above 3.22 pM in 9 out of 15 BAL samples, while only 1 out of 15 BAL samples contained PGE_2_ levels above 3.22 pM after filtered air exposure. On the contrary, PGF_2α_ (also a COX metabolite) had a Youden score of <16.2 pM in BW, which suggested that levels below 16.2 pM in BW were indicative of biodiesel exhaust exposure. Indeed, BW samples from biodiesel exhaust exposure contained levels of PGF_2α_ below 16.2 pM in 11 out of 15 samples, while only four BW samples contained PGF_2α_ levels below 16.2 pM after filtered air exposure.

### Compartment-specific profiles of oxylipins and *N*-acylethanolamines

**Fig. 7:**
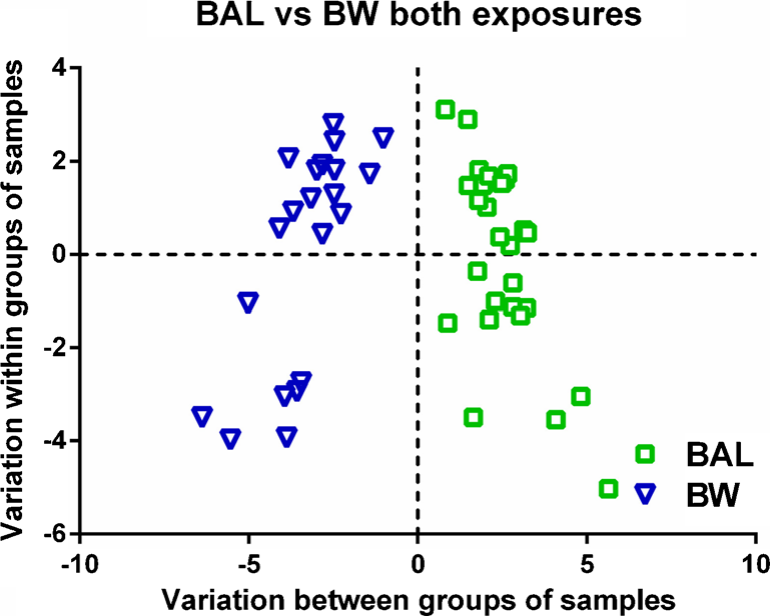
Orthogonal projections to latent structures with discriminant analysis (OPLS-DA) score plot showing the separation between bronchoalveolar lavage (*BAL*) and bronchial wash (*BW*) samples independent of exposure and inclusive of all variables. Model parameters were: 1 predictive and 1 orthogonal component, *R* ^2^ *X* (cum) = 0.425; *R* ^2^ *Y* (cum) = 0.302; *Q* ^2^ (cum) = 0.243; CV-ANOVA = 0.0002

Similar to what was described by Larsson et al. [42], it was possible to group samples by lung compartment in separate clusters of BW and BAL samples obtained by a significant OPLS-DA model based on analytes found in 75% of BW or BAL samples (CV-ANOVA = 0.0002, Fig. 7). This separation occurred for samples collected after both biodiesel exhaust and filtered air exposure. Furthermore, there was a relationship between the OPLS-DA models generated for biodiesel exhaust and filtered air exposure, presented in a shared and unique structure (SUS) plot (Fig. S1, ESM) that provided evidence that the overall, compartment-specific profiles did not change considerably as a consequence of exposure to biodiesel exhaust. SUS plots of filtered air exposure profiles (*y*-axis) against biodiesel exposure profiles (*x*-axis) produced similar characteristic variables for BAL (1st quadrant) and for BW (3rd quadrant) for both types of exposure (Fig. S1, ESM)

**Fig. 8:**
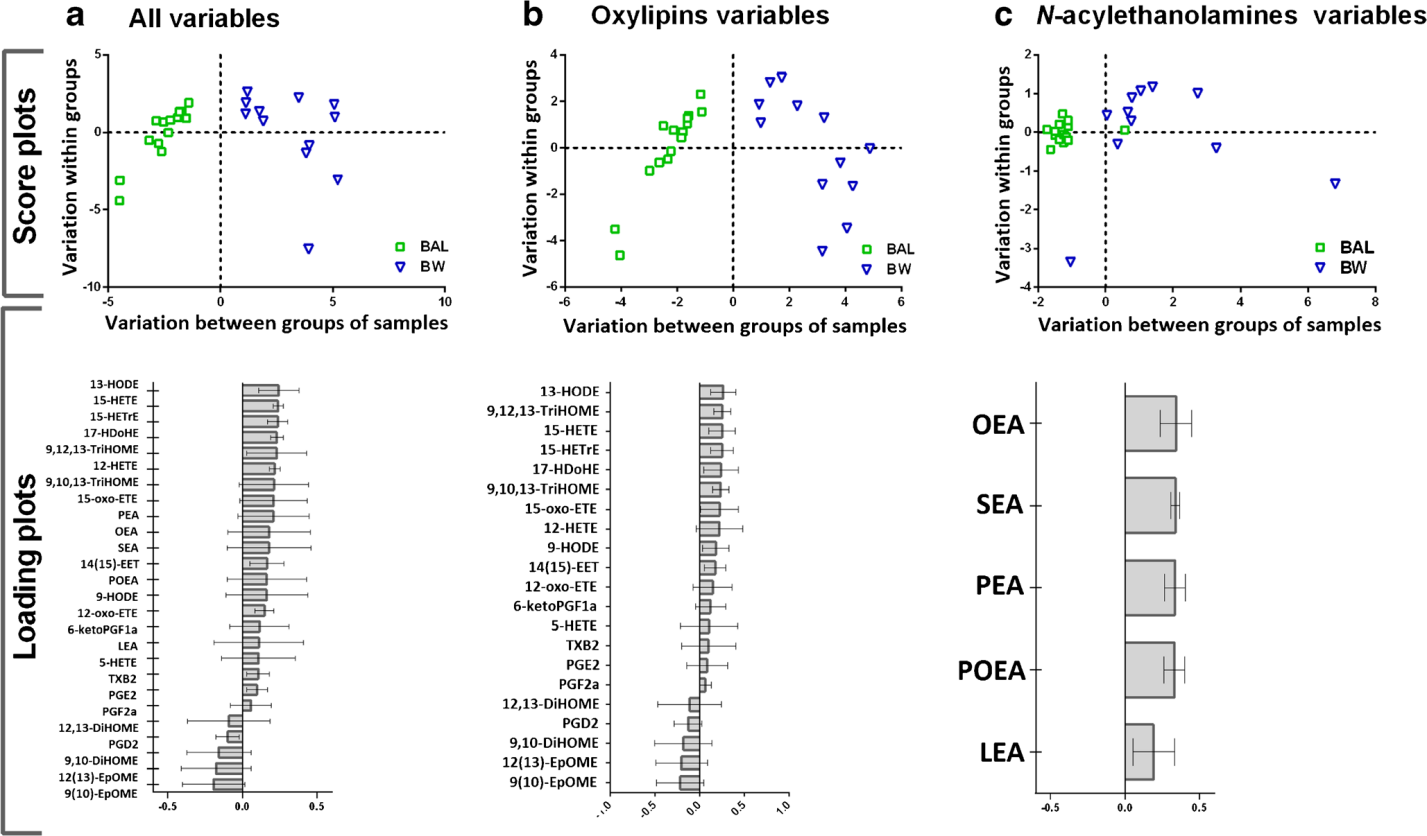
Orthogonal projections to latent structures with discriminant analysis (OPLS-DA) models showing the separation between bronchoalveolar lavage (*BAL*) and bronchial wash (*BW*) samples collected after filtered air exposure comprising: **a** all variables (model parameters: 1 predictive and 1 orthogonal component, *R* ^2^ *X* (cum) = 0578; *R* ^2^ *Y* (cum) = 1; *Q* ^2^ (cum) = 0.69; CV-ANOVA = 2.1 × 10^−5^); **b** oxylipin variables (model parameters: 1 predictive and 1 orthogonal component, *R* ^2^ *X* (cum) = 0.582; *R* ^2^ *Y* (cum) = 1; *Q* ^2^ (cum) = 0.767; CV-ANOVA = 1.1 × 10^−6^); and **c** *N*-acylethanolamine variables (model parameters: 2 predictive and 1 orthogonal components, *R* ^2^ *X* (cum) = 0.578; *R* ^2^ *Y* (cum) = 0.486; *Q* ^2^ (cum) = 0.556; CV-ANOVA = 0.012)

Univariate statistics confirmed the findings that there were significant differences between the BAL and BW concentrations of 15 metabolites, independent of exposure, and 4 metabolites after only one type of exposure (Table 1). The differences between the BW and BAL profiles are of biological importance because they represent different compartments of the lung, an issue that requires further exploration. To that end, the BW and BAL samples from filtered air exposure were inspected by OPLS-DA for all analytes found in >75% of samples included (Fig. 8a), as well as with oxylipins and *N*-acylethanolamines as separate variables (Fig. 8b, c, respectively). The models generated, comprising all variables (CV-ANOVA = 2.1 × 10^−5^), only oxylipins (CV-ANOVA = 1.1 × 10^−6^), or only *N*-acylethanolamines (CV-ANOVA = 0.012), were all significant. Analytes that were responsible for the separation in Fig. 8a were mainly 9(10)-EpOME and 12(13)-EpOME (BAL), as well as 13-HODE and 15-HETE (BW); this finding matches the results from previous studies of healthy individuals [42]. The major *N*-acylethanolamines responsible for the separation were OEA and SEA which were present at higher concentrations in BW than in BAL (Fig. 8c).

Multivariate statistical analysis was also conducted to investigate the overall trends between the two exposures (filtered air and biodiesel exhaust). The ESM provides a description of this analysis. In accordance with the results of the univariate analysis and ROC curve calculations, PGE_2_ was the major contributor to the exposure-dependent separation in an OPLS-DA model of COX metabolites in BAL samples (CV-ANOVA = 0.02, Fig S2, ESM). LOX and CYP products, separately, as well as all detected lipid metabolites investigated in this study, did not produce valid models.

## Discussion

In this study, we explored the responsiveness of bioactive lipid mediators in human airways to an oxidative challenge comprised of controlled exposure to biodiesel exhaust. Biodiesel use is increasing in importance as a renewable carbon dioxide neutral biofuel, and in the present study RME was employed as being among the most common biodiesel fuel in northern Europe. The findings pinpoint a subset of bioactive lipids with diagnostic value for exposure of exhaust derived from a pure RME-biodiesel fuel (B100), which warrants validation in future studies. Further investigations are needed to study differences in effects from biodiesel and conventional petrodiesel exhaust exposure since the exploratory nature of our study poses a limitation and the results cannot be generalized to other biodiesel blends and origin, nor to petrodiesel.

Lung lavage fluids representing proximal vs peripheral lung compartments were collected. The analytes selected for quantification are acknowledged as important for different inflammatory conditions (acute and/or chronic), and a limited number of studies in humans have described a subset of them as being responsive to allergens and air pollution exposure (although not with regard to combustion-derived exhaust exposure) [42–45]. Our study combined a large number of these analytes. However, direct inter-study comparisons of analyte concentrations in lung lavage fluids may be dubious given that variations in for example bronchoscopy protocols must be taken into account.

Interestingly, we detected six *N*-acylethanolamines in both BAL and BW: the well-known CB receptor ligand anandamide (or AEA), as well as LEA, OEA, PEA, POEA, and SEA. While only limited information exists on the prevalence of endocannabinoids, *N*-acylethanolamines, and fatty acid glycerol esters in human lung lavage fluids, studies have shown that eicosanoids and associated oxylipins are present at compartment-dependent concentrations, and that the levels are affected by subway air exposure [42, 44].

In the present study, we aimed to investigate air pollution-induced effects at different levels of the airways, as distinct responses have been reported in previous studies following exposure to air pollutants [18, 46]. One explanation for not always detecting these differences is that changes in the more proximal airways might be masked by the large-scaled addition of alveolar component when all the recovered lavage fluid is pooled. To overcome this problem, we thus used sequential collection of lung lavage fluids using first a small volume of instilled saline solution (resulting in BW), followed by a larger volume (resulting in BAL), as BW is suggested to mainly identify inflammation in the bronchi/airways compared with the large-volume BAL, which has a greater potential to reflect conditions in the alveoli. In a number of previous controlled air pollution exposure studies in healthy humans, this method of differential lavage sampling has been employed. We have previously demonstrated marked neutrophilic inflammation in the proximal airways sampled by endobronchial biopsies and BW following exposure to petrodiesel, however, with no corresponding inflammatory response in BAL fluids, representing the more peripheral and alveolar lung compartments [17, 18, 47–49]. Moreover, clear differences between the responses of the airway and alveolar regions have been identified following controlled exposure to wood smoke [50]. The approach using BW and BAL fluids has also been advocated in previous studies addressing oxylipin profiles in the airways of healthy and asthmatics subjects [42, 44]. While the distinction of proximal (airway) and distal (alveolar) regions using the above-mentioned bronchoscopy technique is not absolute, but is shown to be adequate in previous studies [17, 47–49], these regions display a considerably difference in the composition of airway epithelial cells. This affects the production of inflammatory markers within the airways, i.e., lung surfactant phospholipids secreted by the type II alveolar epithelial cells are exclusively produced in the alveoli [51]. Since the airway inflammatory response to biodiesel exhaust in humans is still unknown, as is the airway region for its main response, the present well-established study protocol was employed, in order to disentangle the regional responses on bioactive lipid mediators following a biodiesel exhaust challenge.

Consistent with these previous studies, the current investigation showed compartmental specificity with regard to both oxylipins and *N*-acylethanolamines. For instance, the LA epoxides 9(10)-EpOME and 12(13)-EpOME were indicative of more distal lung regions (BAL), while 13-HODE and 15-HETE indicated more proximal lung regions (BW). However, individual oxylipins and *N*-acylethanolamines did not display the same response to biodiesel exhaust exposure in BW and BAL. The same number of analytes displayed a significant shift in BAL and BW (three in each, but of different identity). Of these, PGE_2_, 12,13-DiHOME, and 13-HODE were significantly elevated in BAL after biodiesel exhaust exposure, using Bonferroni correction for multiple testing. No metabolite from the BW samples reached this level of statistical power. These findings were supported by AUC values from ROC curve calculations of each metabolite, indicating their diagnostic value.

PGE_2_, a COX metabolite derived from AA, is a well-studied compound [52]. PGE_2_ is usually considered a pro-inflammatory compound, found at elevated levels in various conditions such as tumor growth and pain [22, 53]. However, endogenous PGE_2_ is present at higher concentrations in the lung than in plasma and has anti-inflammatory and bronchodilator properties despite being associated with airway irritation and coughing [54]. In a recent study using a mouse model, Birrell et al. concluded that the anti-inflammatory properties of PGE_2_ are mediated via activation of the EP4 prostanoid receptor, resulting in suppression of airway irritation, supporting a protective effect PGE_2_ in the lung [55]. Bronchial epithelial cell-derived PGE_2_ has also been shown to dampen the reactivity of dendritic cells [56]. However, in a recent paper, it was shown that PGE_2_ induced significant airway microvascular leak in mice and guinea pigs that was mediated via activation of the EP2 and EP4 receptors, implying destructive effects of PGE_2_ [57]. The intricate function of PGE_2_ is only one example of the complexity of an adequate inflammatory response that, besides PGE_2_, also involves other studied eicosanoids and associated bioactive lipid mediators that altogether exhibit coordinated and often opposing actions [22]. Our results suggest that PGE_2_ portrays a protective response in the more distal parts of the lung, where fewer signs of an inflammatory response have been detected than in BW [17, 18, 50]. But further research is needed to test this hypothesis.

Besides PGE_2_, the increased levels of 12,13-DiHOME and 13-HODE in BAL also reached the significance threshold, after correction for multiple testing, in response to biodiesel exhaust exposure. Both metabolites derive from LA, but 12,13-DiHOME is produced through the CYP pathway (containing the downstream soluble epoxide hydrolase enzyme), while 13-HODE is produced via the LOX pathway. Contrary to PGE_2_, these two metabolites have been associated with adverse inflammatory outcomes in the airways. In fact, the elevation of both potentially anti-inflammatory (PGE_2_) and pro-inflammatory (12,13-DiHOME and 13-HODE) lipid mediators in BAL in response to biodiesel exhaust exposure illustrates the complicated interplay between inflammatory lipid mediators, as described above. The metabolite 12,13-DiHOME, known as iso-leukotoxin-diol, is produced in leukocytes [42]. Studies report elevated 12,13-DiHOME levels in exhaled breath condensate from an asthmatic individual following allergen exposure [58] and elevated leukotoxin levels in BAL fluid from subjects with respiratory distress syndrome [59]. The metabolite 13(S)-HODE is involved in allergic responses and airway epithelial injury [60], and specifically targets the receptor PPARγ (which is believed to regulate the underlying inflammation in many airway diseases) [61].

Furthermore, the current study suggests decreased PGF_2α_ and 15-HETE concentrations, and increased 9-HODE concentrations in BW in response to biodiesel exhaust exposure. These metabolites derive from AA and LA; PGF_2α_ is a COX metabolite (in part produced from PGE_2_), while 15-HETE and 9-HODE are LOX metabolites. 15-HETE has antioxidant and pro-resolving properties [62] and was significantly downregulated in BAL fluid in a murine cystic fibrosis model after nanoparticle exposure [63]. Hence, the trend towards decreased levels of 15-HETE in BW may indicate a lack of protection against the harmful effects of biodiesel exhaust exposure in more proximal parts of the lung, where, based on previous petrodiesel studies, more signs of an inflammatory response have been detected in traditional inflammatory markers, compared to BAL [17, 18, 50]. 9-HODE previously demonstrated opposite shifts in response to exposure to subway air, with increased concentrations from healthy subjects and decreased concentrations from asthmatics [44]. Hence, our results showing increased 9-HODE levels in response to biodiesel exhaust exposure corroborated previous findings, although using a different type of air pollution exposure.

## Conclusions

The effect of biodiesel exhaust exposure on human health has long been overlooked in spite of calls for performing studies in this field. Since novel methods for assessing these health effects are desirable, it is attractive to establish profiling techniques to comprehend the complex signaling networks of bioactive lipids that are imperative in coping with inflammatory events. To that end, we used quantitative mass spectrometry profiling protocols to cover a large array of potential bioactive lipids in human lung lavage fluids. Thereby, we quantified multiple oxylipins (including eicosanoids), endocannabinoids, and *N*-acylethanolamines to investigate the responsiveness to RME biodiesel exhaust exposure. The results showed that individual lipids from different lung compartments displayed a change in levels in response to biodiesel exhaust exposure, and a subset was of diagnostic value. Since lipid mediators are important regulators of a wide variety of physiological responses, and since we have shown the responsiveness and diagnostic value of a subset of them to biodiesel exhaust exposure, they might be useful markers of the impact of conventional fuels as well as new, commercially available biofuels, on human health in future investigations.

## Electronic supplementary material

Below is the link to the electronic supplementary material.

ESM 1 (PDF 679 kb)

ESM 2 (XLSX 41 kb)
