# Supplementary material for: Mass spectrometry profiling of oxylipins, endocannabinoids, and N-acylethanolamines in human lung lavage fluids reveals responsiveness of prostaglandin E2 and associated lipid metabolites to biodiesel exhaust exposure
Source: Anal Bioanal Chem. 2017 Feb 24;409(11):2967–80. doi: 10.1007/s00216-017-0243-8 (PMC5366178; doi:10.1007/s00216-017-0243-8)
Supplement: Supplementary file 1 — (PDF 679 kb) [file 216_2017_243_MOESM1_ESM.pdf]

**Analytical and Bioanalytical Chemistry**

**Electronic Supplementary Material**

# Mass spectrometry profiling of oxylipins, endocannabinoids, and *N*-acylethanolamines in human lung lavage fluids reveals responsiveness of prostaglandin E2 and associated lipid metabolites to biodiesel exhaust exposure

Sandra Gouveia-Figueira, Masoumeh Karimpour, Jenny A. Bosson, Anders Blomberg, Jon Unosson, Jamshid Pourazar, Thomas Sandström, Annelie F. Behndig, Malin L. Nording

Additional file available under „Supplementary material"

## Bronchoscopy method

Six hours after the end of the biodiesel exhaust and filtered air exposures respectively, video bronchoscopy was performed to obtain lavage samples. Premedication with 1.0 mg of atropine was given subcutaneously 30 minutes before the procedure. Lidocaine was used for topical anaesthesia. A flexible video bronchoscope (Olympus BF-1T160, Tokyo, Japan) was inserted through the mouth with the subject in the supine position. In a randomised manner, bronchial wash (BW, 2x20 mL) and bronchoalveolar lavage (BAL, 3x60 mL) were carried out on either the lingual or the middle lobe bronchus, using sterile sodium chloride. The aspirates recovered from the installations of BW and BAL were collected into separate siliconised containers and immediately placed in iced water.

## Multivariate statistical analysis

PCA was done to examine the variation and possible clusters with all analytes included (Fig. S3), as well as oxylipins and *N*-acylethanolamines separately (Fig. S4 and Fig. S5, respectively). PCA score plots do not show clear exposure dependence but it is sensitive to type of lung fluid.

OPLS-DA [32] was used to investigate the relationship within i) exposure type and ii) type of sample analyzed (BW vs. BAL). The OPLS-DA model for BAL and BW samples together including all variables was not significant in separating biodiesel exhaust from sham exposure. The same modeling approach was performed for BAL and BW samples separately and including i) all analytes or ii) oxylipins and *N*-acylethanolamines separately. Again no significant model (CV-ANOVA 0.2 – 0.8) was found in separating biodiesel exhaust from sham exposure.

We then selected oxylipin variables according to their enzymatic pathway (i.e. COX, LOX, CYP separately, see Fig. 1 for assignments). OPLS-DA models based on LOX and CYP products did not produce any valid model. However, COX metabolites in BAL resulted in a significant model (CV-ANOVA 0.02, Fig. S2A) separating biodiesel exhaust and sham exposure along the y-axis. The corresponding loading plot is shown in Fig. S2B, representing the influence of each variable on the sample profiles.

**Table S1** Multiple reaction monitoring (MRM) transitions and retention times (RT) for the oxylipins screened for in lung lavage samples (BW and BAL)

| Analyte | Pathway | RT (minutes) | Quantifying MRM transition | | Qualifying MRM transition | |
|---|---|---|---|---|---|---|
| | | | *Precursor ion* | *Product ion* | *Precursor ion* | *Product ion* |
| 6-keto-$PGF_{1\alpha}$ | COX | 6.24 | 369.1 | 163.00 | *n.a* | *n.a* |
| $TXB_2$ | COX | 7.57 | 369.23 | 169.10 | 369.23 | 195.00 |
| 9,12,13-TriHOME | LOX | 8.17 | 329.23 | 211.10 | 329.23 | 229.10 |
| 9,10,13-TriHOME | LOX | 8.34 | 329.23 | 171.00 | 329.23 | 139.10 |
| $PGF_{2\alpha}$ | COX | 8.36 | 353.23 | 193.30 | 353.23 | 211.00 |
| $PGE_2$ | COX | 8.64 | 351.21 | 315.10 | 351.21 | 271.20 |
| $PGD_2$ | COX | 9.12 | 351.21 | 315.20 | 351.21 | 271.10 |
| Resolvin D2 | LOX | 9.45 | 375.21 | 215.10 | 375.21 | 216.10 |
| Resolvin D1 | LOX | 9.86 | 375.21 | 215.10 | 375.21 | 217.10 |
| 5(S)6(R)-$LXA_4$ | LOX | 9.98 | 351.00 | 115.00 | *n.a* | *n.a* |
| 5(S)6(S)-$LXA_4$ | LOX | 10.26 | 351.00 | 115.00 | *n.a* | *n.a* |
| *Trans*-$LTB_4$ | LOX | 12.61 | 335.22 | 195.10 | 335.22 | 317.20 |
| $LTB_4$ | LOX | 12.91 | 335.22 | 195.10 | 335.22 | 317.20 |
| 12,13-DiHOME | CYP | 13.27 | 313.24 | 183.20 | 313.24 | 99.00 |
| 9,10-DiHOME | CYP | 13.72 | 313.20 | 201.00 | 313.20 | 59.10 |
| 14,15-DHET | CYP | 14.03 | 337.24 | 207.00 | 337.24 | 129.20 |
| 11,12-DHET | CYP | 14.73 | 337.24 | 167.10 | *n.a* | *n.a* |
| 8,9-DHET | CYP | 15.30 | 337.24 | 127.20 | *n.a* | *n.a* |
| 5,6-DHET | CYP | 15.69 | 337.24 | 71.00 | 337.24 | 145.10 |
| 12-HEPE | LOX | 16.14 | 317.21 | 179.10 | 317.21 | 299.10 |
| 20-HETE | CYP | 15.76 | 319.23 | 289.20 | 319.23 | 180.10 |
| 13-HODE | LOX | 16.73 | 295.23 | 195.10 | 295.23 | 277.10 |
| 9-HODE | LOX/Auto-ox | 16.89 | 295.23 | 171.20 | 295.23 | 277.20 |
| 15-HETE | LOX | 17.09 | 319.23 | 219.00 | 319.23 | 301.20 |
| 17-HDoHE | LOX | 17.17 | 343.23 | 281.20 | 343.23 | 201.30 |
| 13-oxo-ODE | LOX | 17.20 | 293.21 | 113.10 | 293.21 | 165.20 |
| 15-oxo-ETE | LOX | 17.45 | 317.21 | 113.20 | 317.21 | 273.10 |
| 11-HETE | LOX | 17.50 | 319.23 | 167.20 | *n.a* | *n.a* |
| 12-HETE | LOX | 17.76 | 319.23 | 179.10 | *n.a* | *n.a* |
| 8-HETE | LOX | 17.83 | 319.23 | 155.00 | 319.23 | 301.20 |
| 15-HETrE | LOX | 17.95 | 321.24 | 303.30 | 321.24 | 221.10 |
| 12-oxo-ETE | LOX | 17.95 | 317.21 | 273.30 | 317.21 | 153.20 |
| 9-HETE | LOX | 18.03 | 319.23 | 167.20 | 319.23 | 123.10 |
| 5-HETE | LOX | 18.28 | 319.23 | 115.10 | 319.23 | 301.10 |
| 12(13)-EpOME | CYP | 18.52 | 295.23 | 195.10 | 295.23 | 277.20 |
| 14(15)-EET | CYP | 18.54 | 319.22 | 219.00 | 319.22 | 301.00 |
| 9(10)-EpOME | CYP | 18.72 | 295.23 | 171.20 | *n.a* | *n.a* |
| 5-oxo-ETE | LOX | 19.00 | 317.23 | 203.20 | 317.23 | 59.10 |
| 11(12)-EET | CYP | 18.96 | 319.23 | 167.10 | 319.23 | 301.2 |
| 8(9)-EET | CYP | 19.19 | 319.23 | 69.20 | 319.23 | 123.00 |
| 5(6)-EET | CYP | 19.34 | 319.23 | 191.10 | *n.a* | *n.a* |

*n.a = not applicable due to lack of stable transition*

**Table S2** Multiple reaction monitoring (MRM) transitions and retention times (RT) for the endocannabinoids and related lipids screened for in lung lavage samples (BW and BAL)

| **Analyte** | **RT (minutes)** | **Quantifying MRM transition** | | **Qualifying MRM transition** | |
|---|---|---|---|---|---|
| | | *Precursor ion* | *Product ion* | *Precursor ion* | *Product ion* |
| $PGF_{2\alpha}$-EA | 3.11 | 380.00 | 62.10 | 380.00 | 348.00 |
| $PGE_2$-EA | 3.18 | 378.00 | 62.10 | 396.00 | 62.10 |
| 2-LG | 5.52 | 355.02 | 263.00 | *n.a* | *n.a* |
| EPEA | 5.68 | 346.10 | 62.10 | 346.10 | 201.00 |
| POEA | 6.39 | 298.00 | 62.10 | 298.00 | 281.00 |
| DHEA | 6.45 | 372.00 | 62.10 | 372.00 | 311.00 |
| AEA | 6.78 | 348.10 | 62.10 | 348.10 | 203.00 |
| NAGly | 6.86 | 362.00 | 287.00 | 362.00 | 203.00 |
| LEA | 6.91 | 324.00 | 62.10 | 324.00 | 245.00 |
| 2-AG | 7.59 | 379.10 | 287.00 | 379.10 | 269.00 |
| PEA | 8.55 | 300.00 | 62.10 | 300.00 | 286.00 |
| DEA | 8.68 | 376.00 | 62.10 | 376.00 | 315.00 |
| OEA | 9.12 | 326.00 | 62.10 | 326.00 | 309.00 |
| SEA | 13.2 | 328.10 | 62.10 | 328.10 | 311.00 |

*n.a = not applicable due to lack of stable transition*

**Table S3** Native standards and corresponding internal standards

| **Internal standard** | **Native standard** |
|---|---|
| *Endocannabinoids and related lipids* | |
| 2-AG-$d_8$ | 2-AG; 2-LG |
| AEA-$d_4$ | AEA |
| OEA-$d_4$ | OEA, EPEA, NAGly, POEA, DHEA |
| PEA-$d_4$ | PEA, DEA, LEA |
| SEA-$d_3$ | SEA |
| $PGF_{2\alpha}$-EA-$d_4$ | $PGF_{2\alpha}$-EA |
| $PGE_2$-EA-$d_4$ | $PGE_2$-EA |
| *Oxylipins* | |
| $TXB_2$-$d_4$ | $TXB_2$, 9,12,13-TriHOME, 9,10,13-TriHOME, |
| 12(13)-DIHOME-$d_4$ | 12(13)-DiHOME, 9(10)-DiHOME, 14,15-DHET, 11,12-DHET, 8,9-DHET, 5,6-DHET, |
| 12(13)-EPOME-$d_4$ | 12(13)-EPOME, 9(10)-EPOME |
| 9(S)-HODE-$d_4$ | 13-HODE, 9(S)-HODE, 17(R)-HDoHE, 13-oxo-ODE, 15-oxo-ETE, 9-oxo-ODE, 15(S)-HETrE, 12-oxo-ETE, 5-oxo-ETE |
| 5(S)-HETE-$d_8$ | 5-HETE, 12-HETE, 8-HETE, 9-HETE, 14(15)-EET, 11(12)-EET, 8(9)-EET, 5(6)-EET |
| 20-HETE-$d_6$ | 12(S)-HEPE, 20-HETE, 15-HETE, 11-HETE |
| $PGE_2$-$d_4$ | $PGF_{2\alpha}$, $PGE_2$, Resolvin $D_1$, Resolvin $D_2$, $LTB_4$ |
| $PGD_2$-$d_4$ | $PGD_2$ |

**Table S4** Concentrations (ng/mL) used for calibration curves of endocannabinoids and related lipids. Stock solutions for all standards were weekly prepared and stored in methanol at -80 °C. Each native standard stock solution was diluted with methanol at ten different calibration levels (S1 – S10) and stored at -80 °C

| | Standard concentration (ng/mL) | | | | |
|---|---|---|---|---|---|
| | 2-AG, 2-LG | POEA, LEA, AEA, DHEA, NAGly, DEA, EPEA | OEA, PEA | SEA | $PGF_{2\alpha}$-EA, $PGE_2$-EA |
| **S1** | 83.3 | 4.17 | 2.08 | 6.94 | 0.67 |
| **S2** | 41.6 | 2.08 | 1.04 | 3.47 | 0.33 |
| **S3** | 20.8 | 1.04 | 0.52 | 1.74 | 0.17 |
| **S4** | 10.4 | 0.52 | 0.26 | 0.87 | 0.08 |
| **S5** | 5.21 | 0.26 | 0.13 | 0.43 | 0.04 |
| **S6** | 2.60 | 0.13 | 0.07 | 0.22 | 0.02 |
| **S7** | 1.30 | 0.07 | 0.03 | 0.11 | 0.01 |
| **S8** | 0.26 | 0.01 | 0.01 | 0.02 | 0.01 |
| **S9** | 0.05 | 0.003 | 0.0013 | 0.004 | 0.001 |
| **S10** | 0.01 | 0.0003 | 0.0001 | 0.0004 | 0.0002 |

**Table S5** Concentrations (pg/mL) used for calibration curves of oxylipins. Stock solutions for all standards were monthly prepared and stored in methanol at -80 °C. Each native standard stock solution was diluted with methanol at ten different calibration levels (S1 – S10) and stored at -80 °C

| | **Standard concentration (pg/mL)** |
|---|---|
| **S1** | 16450 |
| **S2** | 8220 |
| **S3** | 4110 |
| **S4** | 2060 |
| **S5** | 1030 |
| **S6** | 514 |
| **S7** | 257 |
| **S8** | 129 |
| **S9** | 64 |
| **S10** | 37 |

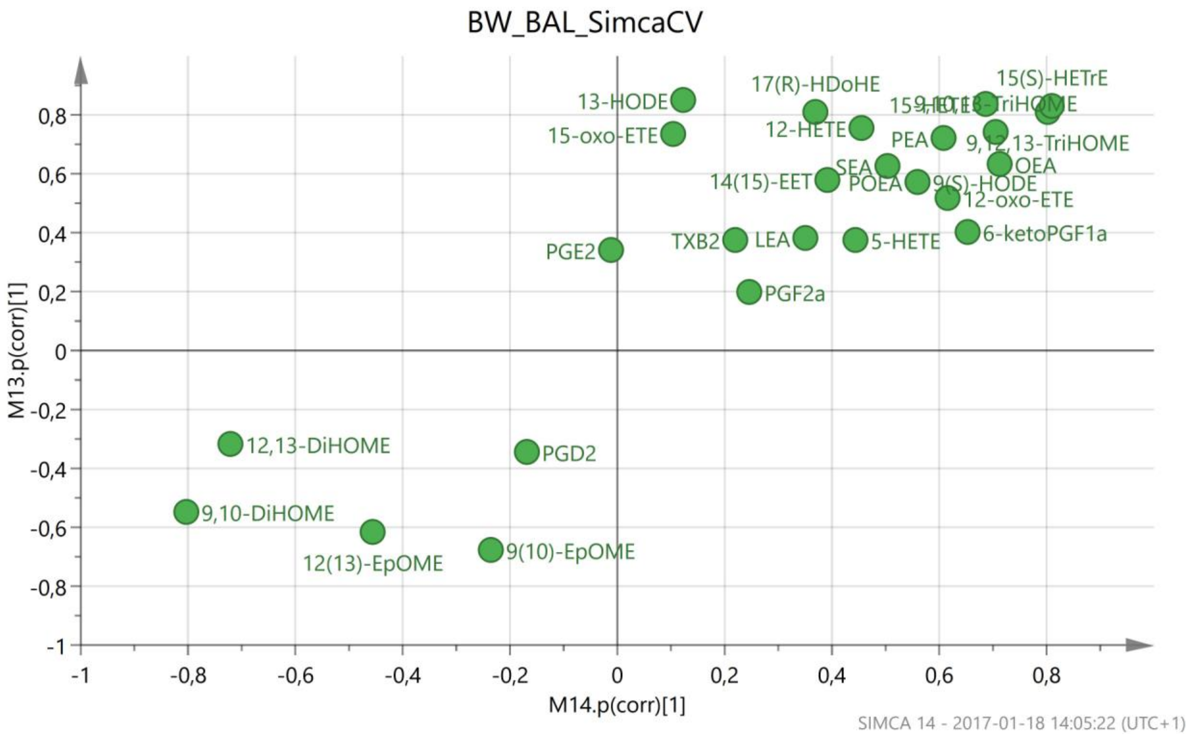


**Fig. S1** OPLS-DA correlation loadings from the biodiesel exhaust (X axis) and sham exposure (Y axis) models plotted against each other in a SUS plot

A

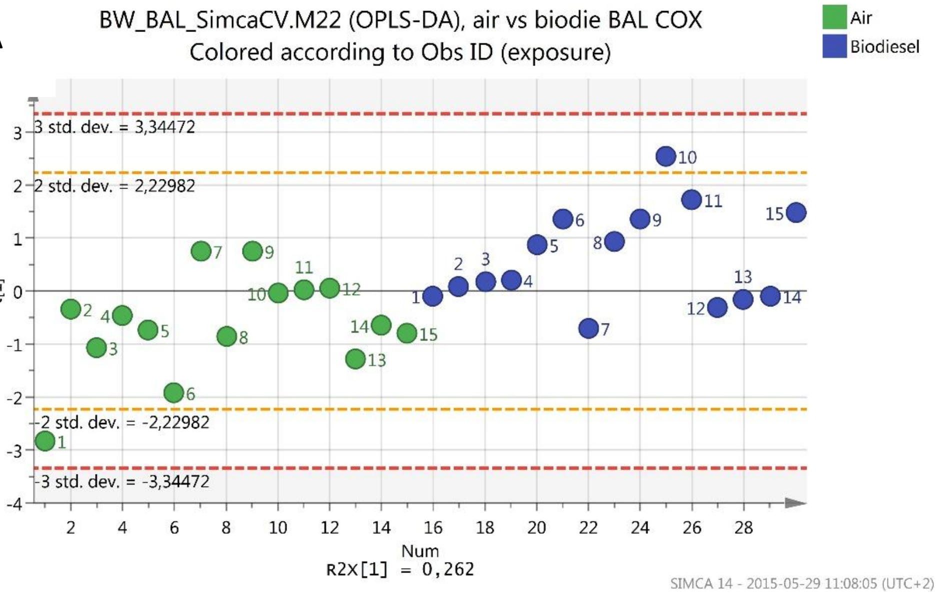


B

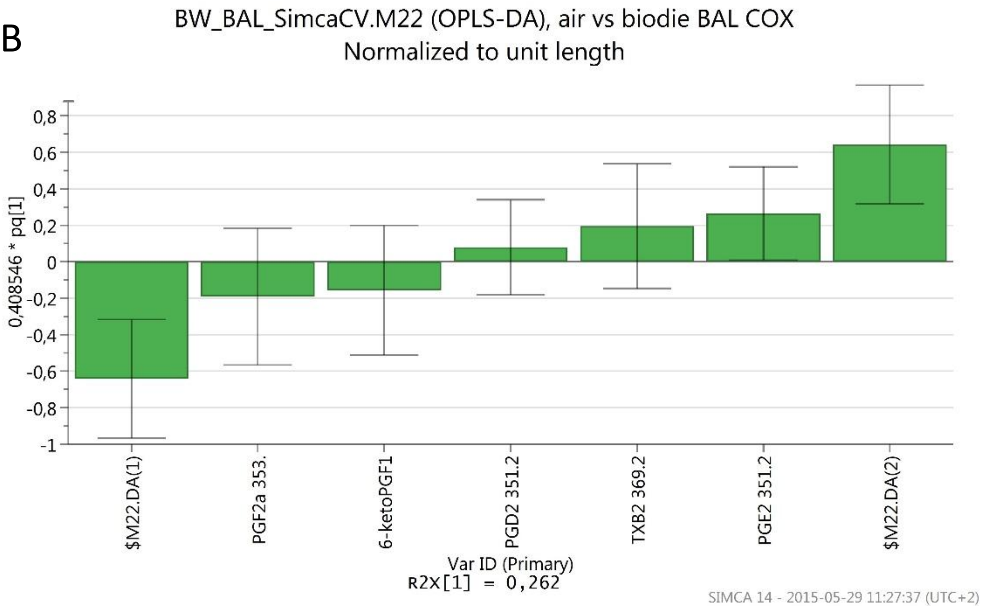


**Figure S2** Oxylipin profile containing analytes from the COX pathway separated by exposure using OPLS-DA: score plot (A); loading plot (B). Model assessment parameters were: 1 predictive component; p-value calculated by CV-ANOVA: 0.02, total systematic variation among the metabolites captured by the model (R2X): 0.262, total systematic variation between the exposures captured by the model (R2Y): 1, predictive ability of the model (Q2): 0.23

A

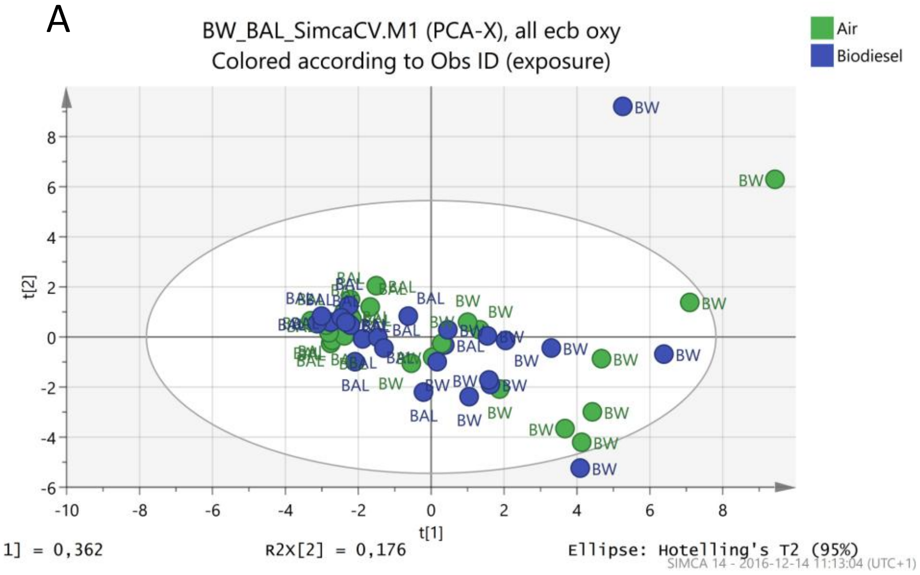


B

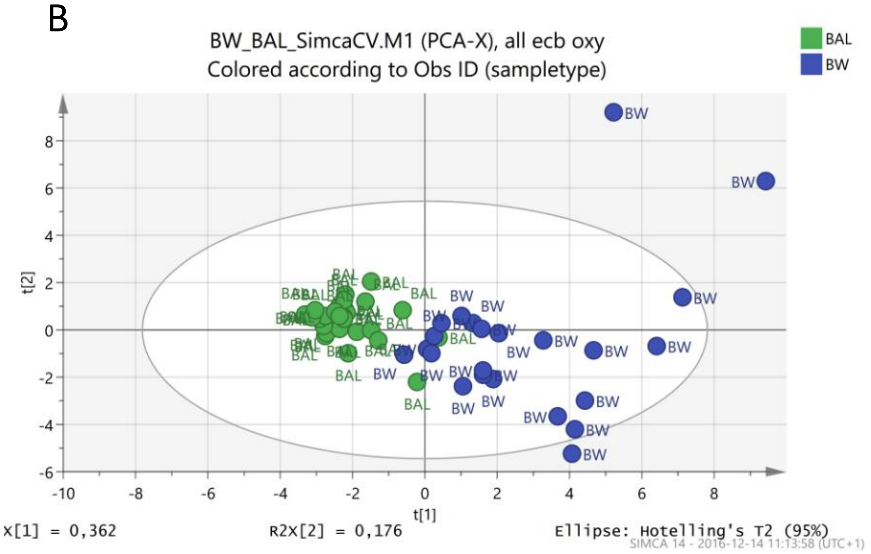


**Fig. S3** PCA score plot using all analytes found in >75% BW or BAL. Coloring representing exposure dependency with air exposure samples in green and biodiesel samples in blue (A); and separation between BAL (green) and BW (blue) samples (B). Model parameters: R2X (cum) 0.656; Q2(cum) 0.371

A

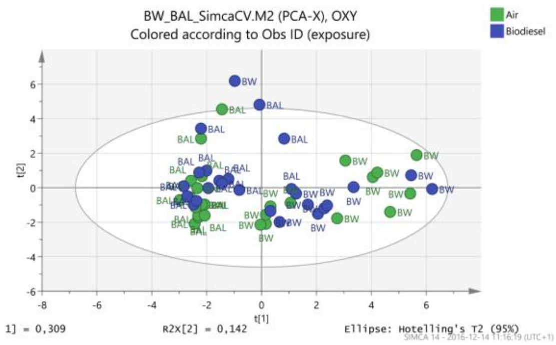


B

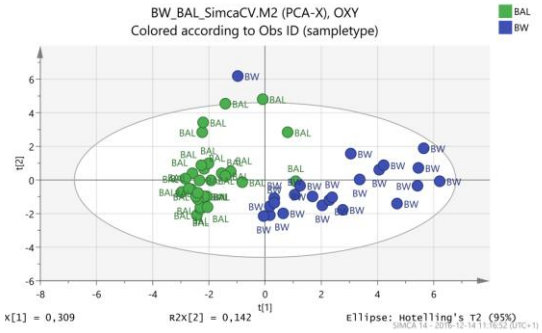


**Fig. S4** PCA score plot using oxylipin variables found in in >75% BW or BAL. Coloring representing exposure dependency with green representing air exposure samples and blue biodiesel samples (A); and separation between BAL (green) and BW (blue) samples (B). Model parameters: R2X(cum) 0.451, Q2 (cum) 0.197

A

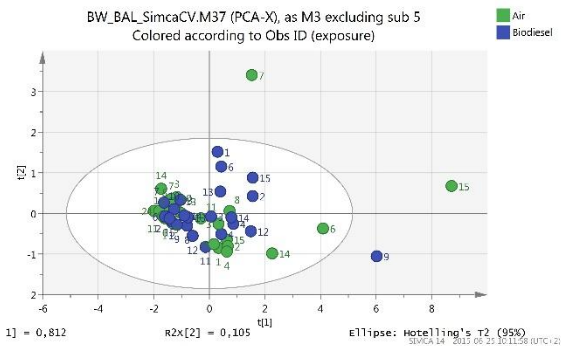


B

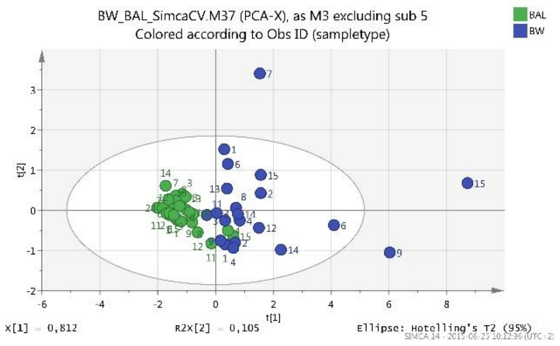


**Fig. S5** PCA score plot using *N*-acylethanolamine variables found in in >75% BW or BAL. Coloring representing exposure dependency with air exposure samples in green and biodiesel samples in blue (A); and separation between BAL (green) and BW (blue) samples (B). PCA model parameters: R2X(cum) 0.92, Q2 (cum) 0.653
